# A prognostic model of idiopathic pulmonary fibrosis constructed based on macrophage and mitochondria-related genes

**DOI:** 10.1186/s12890-024-02976-0

**Published:** 2024-04-12

**Authors:** Yu Bao, Shiyuan Yang, Hailan Zhao, Yezhen Wang, Ke Li, Xue Liu, Wei Zhang, Xue Zhu

**Affiliations:** 1 First School of Clinical Medicine, Shandong University of Traditional Chinese Medicine, Shandong, China; 2 Shandong University of Traditional Chinese Medicine Affiliated Hospital, Shandong, China

**Keywords:** Mitochondria, Macrophage, Idiopathic pulmonary fibrosis, Key genes

## Abstract

**Background:**

Studies have shown that mitochondrial function and macrophages may play a role in the development of idiopathic pulmonary fibrosis (IPF). However, the understanding of the interactions and specific mechanisms between mitochondrial function and macrophages in pulmonary fibrosis is still very limited.

**Methods:**

To construct a prognostic model for IPF based on Macrophage- related genes (MaRGs) and Mitochondria-related genes (MitoRGs), differential analysis was performed to achieve differentially expressed genes (DEGs) between IPF and Control groups in the GSE28042 dataset. Then, MitoRGs, MaRGs and DEGs were overlapped to screen out the signature genes. The univariate Cox analysis and the least absolute shrinkage and selection operator (LASSO) algorithm were implemented to achieve key genes. Furthermore, the independent prognostic analysis was employed. The ingenuity pathway analysis (IPA) was employed to further understand the molecular mechanisms of key genes.Next, the immune infiltration analysis was implemented to identify differential immune cells between two risk subgroups.

**Results:**

There were 4791 DEGs between IPF and Control groups. Furthermore, 26 signature genes were achieved by the intersection processing. Three key genes including *ALDH2, MCL1*, and *BCL2A1* were achieved, and the risk model based on the key genes was created. In addition, a nomogram for survival forecasting of IPF patients was created based on riskScore, Age, and Gender, and we found that key genes were associated with classical pathways including ‘Apoptosis Signaling’, ‘PI3K/AKT Signaling’, and so on. Next, two differential immune cells including Monocytes and CD8 T cells were identified between two risk subgroups. Moreover, we found that MIR29B2CHG and hsa-mir-1-3p could regulate the expression of *ALDH2*.

**Conclusion:**

We achieved 3 key genes including *ALDH2*, *MCL1*,, and *BCL2A1* associated with IPF, providing a new theoretical basis for clinical treatment of IPF.

**Supplementary Information:**

The online version contains supplementary material available at 10.1186/s12890-024-02976-0.

## Introduction

Idiopathic Pulmonary Fibrosis (IPF) is a chronic, progressive, and highly fatal interstitial lung disease characterized by progressive dyspnea and irreversible decline in lung function [1], which seriously threatens patients’ quality of life and survival time [2]. IPF is variable, unpredictable and significantly heterogeneous, and the rate of progression in a single patient in the short term is difficult to predict [3]. Repeated damage and dysrepair of alveolar epithelial cells cause pulmonary fibrosis, including uncontrolled proliferation of lung fibroblasts and excessive deposition of extracellular matrix proteins in the interstitial space, leading to irreversible changes in lung parenchyma and ultimately IPF [4]. The exact mechanism of pulmonary fibrosis involves many theories, such as excessive epithelial damage repair, cellular senescence and mitochondrial dysfunction, insufficient autophagy, epithelial-mesenchymal transformation (EMT), activation of macrophage subpopulation, and telomere shortening [5, 6]. At present, the only anti-fibrosis drugs approved by the FDA are pirfenidone and Nintedanib, both of which have obvious drug toxicity, and the treatment of IPF remains to be explored [7, 8]. Key genes involve many aspects such as immunity and genetics, and can be used to evaluate physiological and pathological processes and responses to drug intervention from an objective perspective. Exploring the pathogenesis of IPF from the level of key genes is helpful for the diagnosis, treatment and prognosis evaluation of the disease [9, 10].

Mitochondria are membrane-closed organelles with independent circular genomes in eukaryotic cells [11]. Mitochondria can not only provide energy for cells by producing ATP through oxidative phosphorylation, but also play an important role in many processes such as apoptosis, signal transduction and substance metabolism [12]. Mitochondria undergo fission and fusion, destroying the original physiological functions and leading to cellular senescence [13]. Mitochondrial autophagy is an important way to maintain functional homeostasis and is closely related to pulmonary fibrosis [14]. Animal experiments have shown that p53 and p21 activate age-related pathways and promote fibrosis [15]. The pathological results of the lung tissues of IPF patients also showed that the expression of telomere-associated DDR sites (TAFs), p16 and other aging markers was increased [16]. Studies have shown that the expression of mitochondrial DNA is negatively correlated with the survival of IPF patients, and therefore, mitochondrial DNA may be a biomarker for predicting acute exacerbation and progression of IPF [17]. In summary, mitochondria are closely related to the occurrence and development of IPF.

Macrophages are an important part of innate immunity. They exist in different tissues as alveolar macrophages, microglia and Kupfer cells. They play an important role in maintaining the balance of the environment in tissues and regulating immunity. Depending on their environment, they can be polarized into different subtypes such as M1 macrophages (also known as classical activated macrophages) and M2 macrophages (also known as alternative activated macrophages) [18]. Both M1 and M2 macrophages are involved in the pathogenesis of pulmonary fibrosis. M1 plays a major pro-inflammatory role in the early inflammatory stage. Subsequently, M2 increases, sustained damage repair and reduction of inflammatory response through various signaling pathways such as TGF-β1/Smad2/3, leading to pulmonary fibrosis [19]. Studies have shown that IPF is related to apoptosis resistance of M2 macrophages [20, 21]. Mitochondrial autophagy, fission, fusion and other dysfunction exist in pulmonary fibrosis [22]. The absence of miR-33 in alveolar macrophages can stabilize the structure and increase the number of mitochondria, thus improving pulmonary fibrosis. Akt1-mediated mitochondrial autophagy can promote the apoptotic resistance of alveolar macrophages and further aggravate pulmonary fibrosis [14]. The above illustrates the mechanisms associated with mitochondrial function and apoptosis and apoptosis and macrophages in IPF. Therefore, further study of the relationship between mitochondria, alveolar macrophages and IPF may enrich the pathogenesis of IPF and contribute to diagnosis and treatment.

Therefore, in this study, genes related to mitochondrial function and macrophages were first obtained, and bioinformatics methods such as univariate Cox and least absolute shrinkage and selection operator (LASSO) regression analysis were used to construct prognostic risk models and search for key genes of IPF to better predict the disease progression and prognosis of patients.

## Materials and methods

### Data sources

The gene expression and sample clinical information data of IPF were achieved by the Gene Expression Omnibus (GEO) online database. The GSE28042 dataset contained 75 IPF samples and 19 Control samples, and these samples were utilized for training set. Moreover, the GSE27957 dataset had 45 IPF samples with clinical information, and it was utilized as validation set. All the samples in GSE28042 and GSE27957 datasets were peripheral blood mononuclear cell (PBMC) samples [23]. The supplementary Tables 1–2 listed details of the clinical characteristics of IPF patients in GSE28042 and GSE27957. In addition, 1136 Mitochondria-related genes (MitoRGs) were achieved through the MitoCarta3.0 online database (http://www.broadinstitute.org/mitocarta). Besides, we acquired 3201 Macrophage-related genes (MaRGs) by the GeneCards online database (https://www.genecards.org/) (Score > 2.5).

### Screening of signature genes

In our study, we used the Benjamini-Hochberg (BH) correction and adjusted the p-value by controlling for the false discovery rate (FDR). To explore the mechanism of IPF pathogenesis and to find genes associated with IPF pathogenesis we performed differential expression analysis. DEGs between IPF and Control groups were acquired by the limma (v 3.50.1) package [24].(p.adjust < 0.05). A heat map and a volcano map of DEGs were plotted by pheatmap (v 1.0.12) and ggplot2 (v 3.3.5) [25]packages, respectively. Furthermore, 1136 MitoRGs, 3201 MaRGs and DEGs were overlapped to screen out the signature genes. For further exploring the biological functions and signaling pathways involved in the signature genes, the Gene Ontology (GO) and Kyoto Encyclopedia of Genes and Genomes (KEGG) enrichment analyses (p.adjust < 0.05) were employed by the clusterProfiler (v 4.2.2) package [26]. In addition, for further understanding the potential interactions of the signature genes, a Protein Protein Interaction (PPI) network was established using the STRING online (https://string-db.org/) database (minimum required interaction score = 0.4).

### Construction and verification of risk model

According to above signature genes, the univariate Cox analysis was performed to identify the candidate genes that related to prognosis of IPF (*p* < 0.05). Furthermore, the LASSO algorithm was implemented to achieve key genes. In addition, we compared differences in key genes between live and dead samples. According to the expression of above key genes, the risk model was created, and the samples in the GSE28042 and GSE27957 datasets were classified into the high- and low-risk groups using the optimum cut-off value of the risk score (risk score = $\sum _{1}^{\text{n}}\text{c}\text{o}\text{e}\text{f}\left({\text{g}\text{e}\text{n}\text{e}}_{\text{i}}\right)\text{*}expression\left({\text{g}\text{e}\text{n}\text{e}}_{\text{i}}\right)$.) respectively. In addition, the Kaplan-Meier (K-M) survival curves and the ROC curves (1-, 2- and 3-year) were plotted, respectively.

### Independent prognostic analysis

In order to construct a prognostic model for clinical survival prediction of IPF patients, riskScore and clinical characteristics (Age and Gender) were combined, then univariate Cox and multivariate Cox analyses were conducted to achieve the independent prognostic factors. Furthermore, a nomogram for forecasting survival rates of the IPF patients (1-, 2- and 3-year) was created. Moreover, the calibration curve was employed to verify the validity of the above nomogram.

### Functional enrichment analysis

In our study, the Gene Set Enrichment Analysis (GSEA) was conducted on all genes (High-risk vs. Low-risk groups) in the GSE28042 dataset (p.adj < 0.05 and|NES| > 1). Furthermore, to further understand the molecular mechanism of key genes, the classical signaling pathway analysis was performed by the ingenuity pathway analysis (IPA) to explore the signaling pathways that key genes were mainly involved in (p.value < 0.05). Subsequently, we selected the largest|z-score| signaling pathway for further displaying the signal pathway transduction process.

### The immune infiltration analysis

For evaluating the degree of the immune cell infiltration, the CIBERSORT algorithm was performed in GSE28042 dataset and we took LM22 as the signature [27]. Meanwhile, immune cells that did not exist in 75% samples were excluded. Subsequently, the Wilcoxon test was implemented to analyze differential immune infiltrating cells between the two risk subgroups. The IPF samples were classified into high- and low-expression groups based on the median expression of key genes. The differences in the differential immune cells between the two expression subgroups of key genes were analyzed. Moreover, the relationships between biomarker and differential immune cells were computed (|cor| > 0.3, *p* < 0.05).

### Regulatory network and the drug prediction

To create the regulatory network, the miRNAs and long noncoding RNA (lncRNA) genes were forecasted using the miRNet online database (www.mirnet.ca). In addition, for exploring potential drugs for the treatment of IPF, the potential drugs for key genes were acquired based on the DGIdb database (www.dgidb.org), and a biomarker-drug network was created. Besides, all the networks were visualized using the cytoscape software [28].

### RT-qPCR

The blood samples were gained from the 5 IPF patients in Affiliated Hospital of Shandong University of Traditiona. And the blood samples obtained from 5 healthy individuals were utilized as Control samples. The blood samples were acquired from the samples to perform RT-qPCR. This study was approved by Ethics Committee of the Affiliated Hospital of Shandong University of Traditional Chinese Medicine. All patients had signed an informed consent form. The expression of the four key genes was further validated via RT-qPCR. Total RNA of 20 samples were extracted using TRIzol (Ambion, Austin, USA) according to the manufacturer’s guidance. Reverse transcription of total RNA to cDNA was carried out by using SureScript-First-strand-cDNA-synthesis-kit (Servicebio, Wuhan, China) based on the manufacturer’s instructions. RT-qPCR was performed utilizing the 2xUniversal Blue SYBR Green qPCR Master Mix (Servicebio, Wuhan, China). The primer sequences for PCR were shown in Table 1. GAPDH was as an internal reference gene. The 2^−ΔΔCt^ method was utilized to calculate the expression of key genes [29].

**Table 1:** Sequence of primers used in the RT-qPCR experiments

Gene name	Primer sequences
ALDH2-F	GCATGGACGCATCACACAG
ALDH2-R	TTGCCATTGTCCAGGGTCTC
MCL1-F	TTGCCATTGTCCAGGGTCTC
MCL1-R	AGGTTGCTAGGGTGCAACTC
BCL2A1-F	AGTGCTACAAAATGTTGCGTTC
BCL2A1-R	GGCAATTTGCTGTCGTAGAAGTT
GAPDH-F	CGAAGGTGGAGTCAACGGATTT
GAPDH-R	ATGGGTGGAATCATATTGGAAC

## Results

### A total of 26 signature genes were acquired

**Fig. 1:**
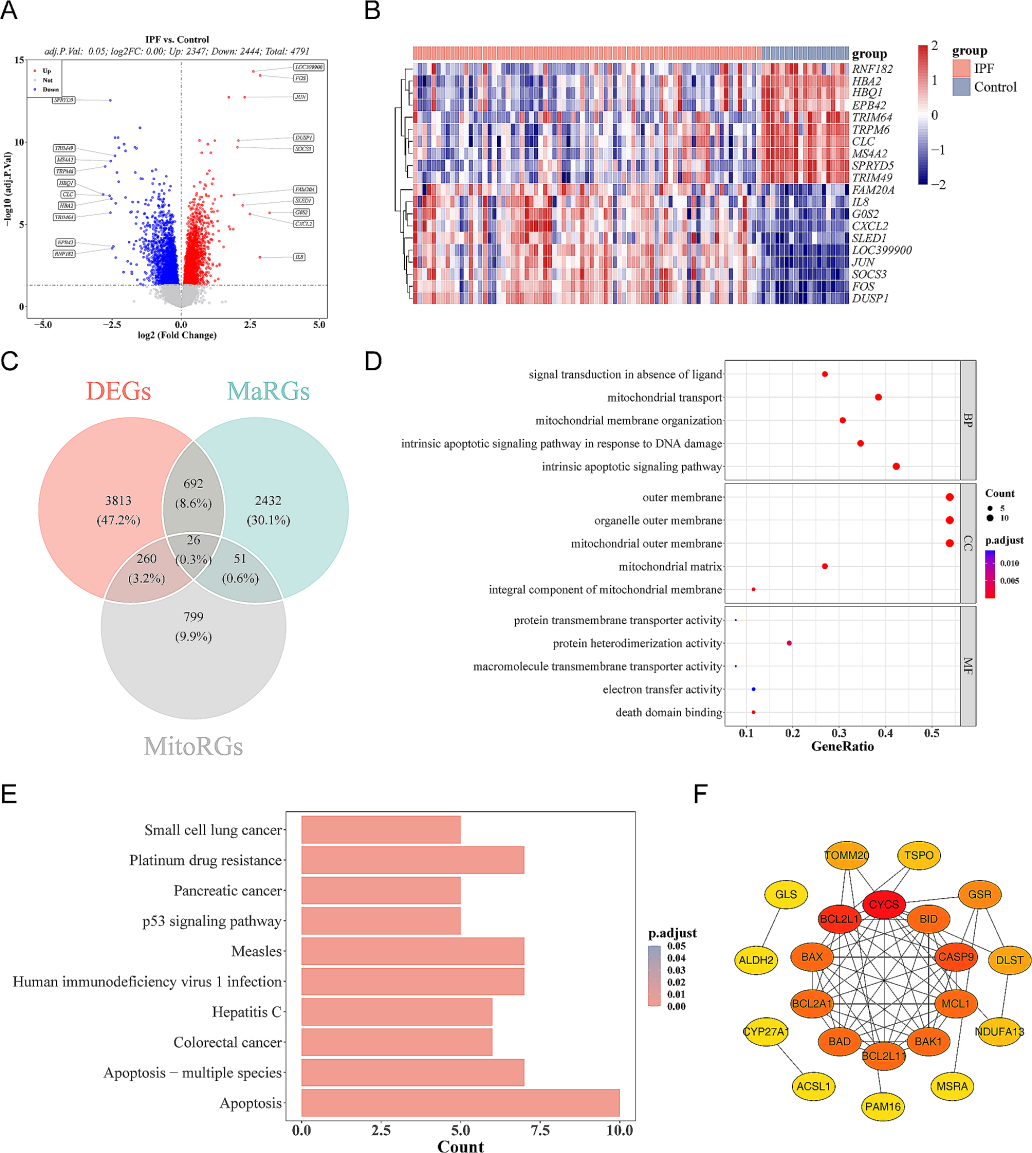
Identification of 26 signature genes. The BH correction was used and the p-value was adjusted by controlling the FDR. **(A)** The volcano plot showed 2347 up-regulated (red) and 2444 down-regulated genes (blue) in the GSE28042 dataset (|log_2_FC|>0, p.adj < 0.05). **(B)** The heatmap of the Idiopathic Pulmonary Fibrosis (IPF)-associated top 10 up- and down-regulated differentially expressed genes (DEGs). **(C)** The venn diagram of DEGs, Macrophage-related genes (MaRGs), and Mitochondria-related genes (MitoRGs). **(D)** The bubble plot of the the top 5 Gene Ontology (GO) Biological Process (BP) terms, GO Cellular Components (CC) terms, GO Molecular Function (MF) terms were enriched for 26 signature genes. **(E)** The column plot of the top 10 Kyoto Encyclopedia of Genes and Genomes (KEGG) pathways were enriched for 26 signature genes. **(F)** The Protein Protein Interaction (PPI) network including 26 signature genes. Nodes represent genes and colors represent Degree values, the redder the value, the stronger the gene’s role relationship in the network

In the GSE28042 dataset, there were 4791 DEGs between IPF and Control groups (Fig. 1A, Supplementary Table 3). The expression heat map of the IPF-associated top 10 up- and down-regulated DEGs was shown in Fig. 1B. Subsequently, 26 signature genes were achieved by the cross-processing (Fig. 1C). According to the functional enrichment analysis, 26 signature genes were mainly associated with ‘signal transduction in absence of ligand’, ‘outer membrane’, ‘protein transmembrane transporter activity’ etc. GO items, and KEGG pathways such as ‘Apoptosis’, ‘p53 signaling pathway’, ‘Apoptosis-multiple species’ and so on (Fig. 1D-E, Supplementary Tables 4–5). Besides, the PPI network including 26 signature genes was established, there were interactions between 21 signature genes, and there was a relatively strong interaction between CYCS and BCL2L1 (Fig. 1F).

### Three key genes were acquired

**Fig. 2:**
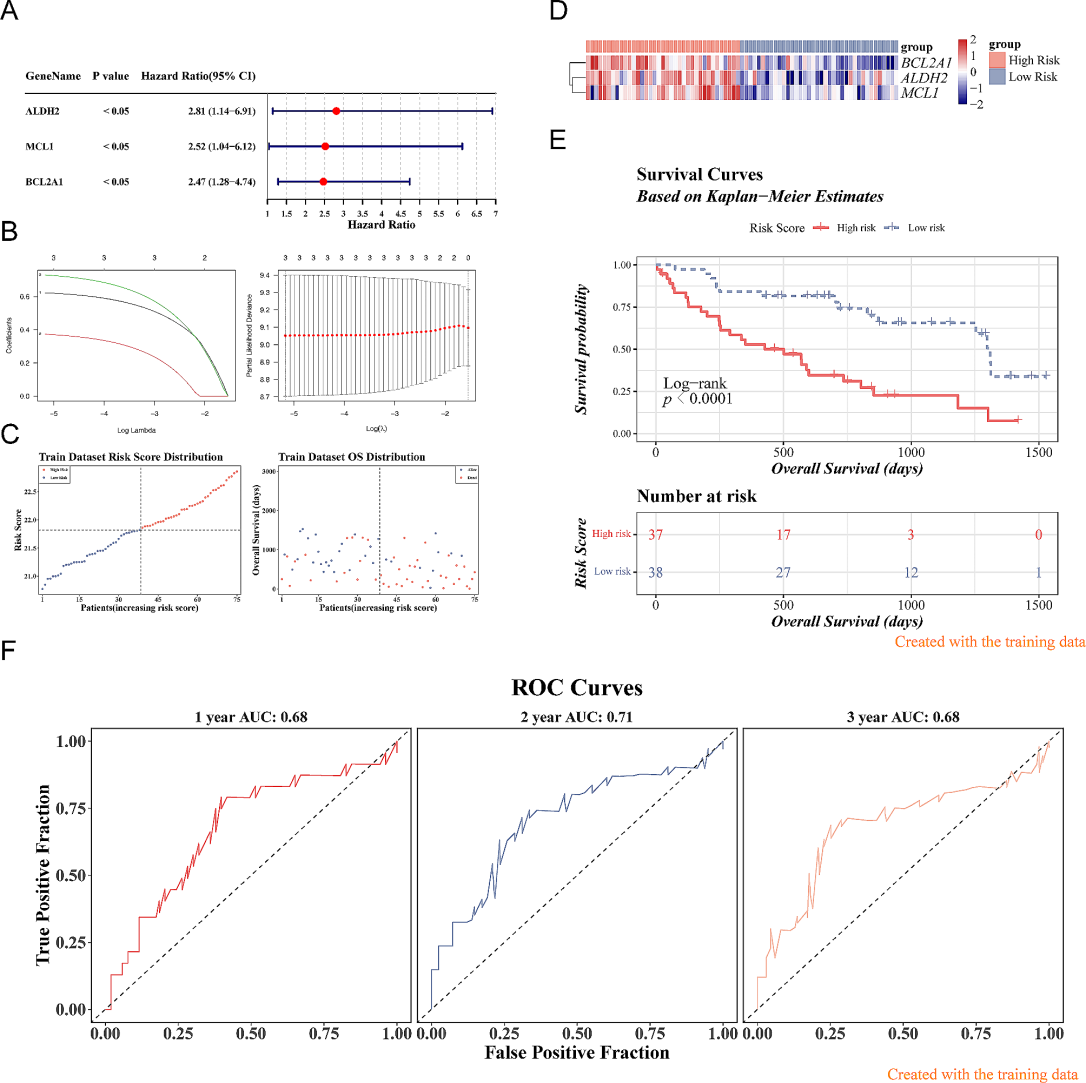
Construction of the risk model based on 3 key genes. **(A)** The forest plot of the univariate Cox analyses of the 3 key genes. **(**p-value < 0.05) **(B)** Least absolute shrinkage and selection operator (LASSO) analysis of the 3 key genes with minimum lambda value. **(C**-**F)** The risk score **(C)**, heat map **(D)**, Kaplan-Meier (K-M) survival **(E)**, and time-dependent receiver operating characteristic (ROC) curves of overall surviva (OS) **(F)** in the GSE28042 dataset. The area under curve (AUC) was assessed at 1, 2 and 3 years

**Fig. 3:**
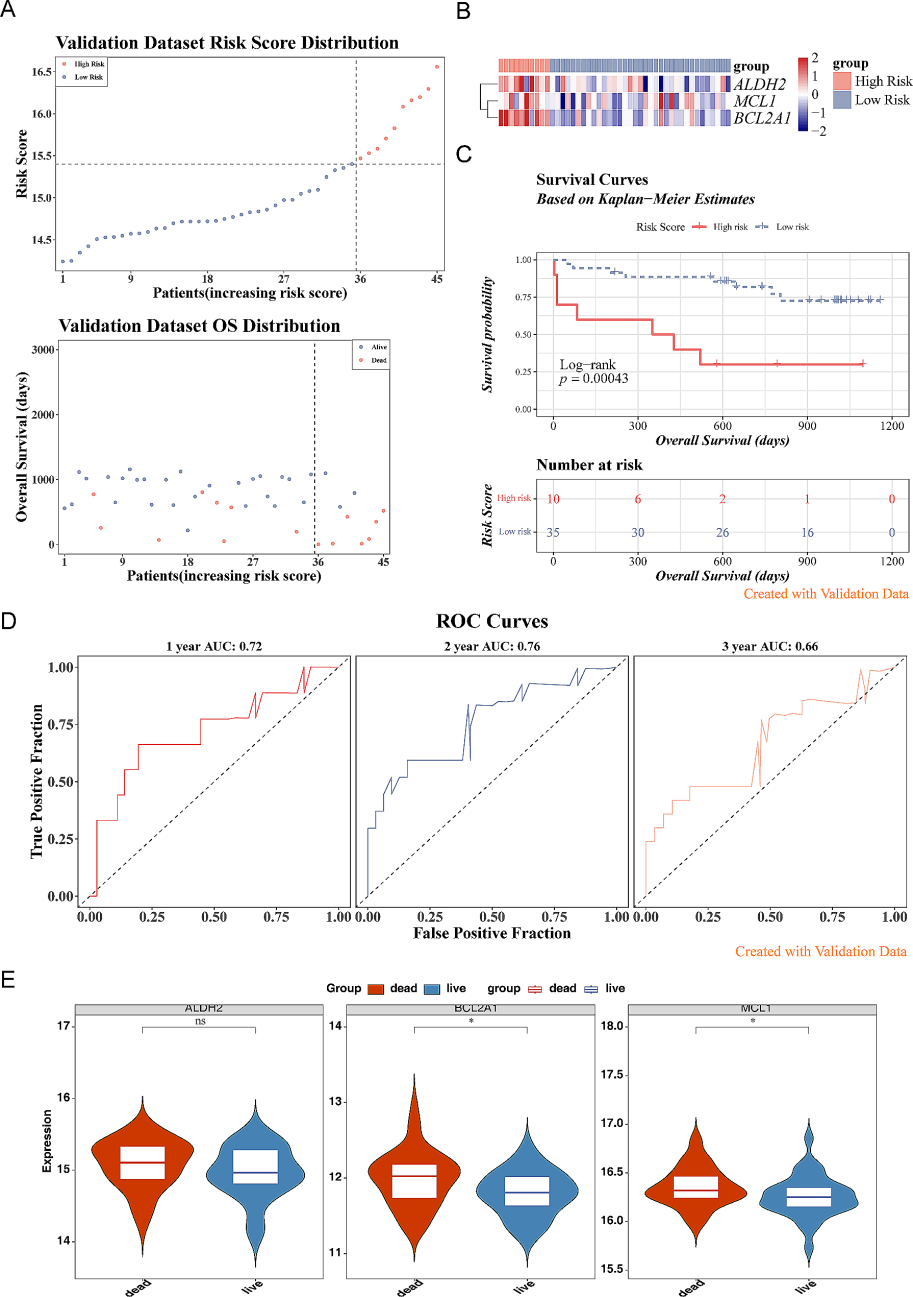
Validation of the risk model based on 3 key genes. **(A**-**D)** The risk score **(A)**, heat map **(B)**, K-M survival **(C)**, and time-dependent ROC curves of OS **(D)** in the GSE27957 dataset. The AUC was assessed at 1, 2 and 3 years. **(E)** Expression of key genes in live and death groups

A total of 3 prognostic candidate genes including *ALDH2, MCL1*,and *BCL2A1* were identified by the univariate Cox analysis (Fig. 2A). Furthermore, there were 3 key genes (*ALDH2*, *MCL1*, and *BCL2A1*) sifted out by the LASSO algorithm (Fig. 2B). In the GSE28042 dataset, IPF samples were classified into high- and low-risk groups based on the optimal cut-off value of risk scores that equal to 21.8207, with the increase of risk score, the number of the dead patients also increased. Moreover, we found that the expression of *ALDH2*, *MCL1*, and *BCL2A1* were downregulated in the low-risk group (Fig. 2C-D). In addition, it was found that there was a distinct difference in survival between these two subgroups (*p* < 0.0001), moreover, the survival rate of the high-risk group was significant decreased (Fig. 2E). The area under curve (AUC) values (1-, 2-, and 3-year) were all above 0.65, it demonstrated that the model had a favorable prediction accuracy and favorable model performance (Fig. 2F). Besides, we verified the risk model in the GSE27957 dataset, moreover, we found that the results were consistent with the GSE28042 dataset (Fig. 3A-D). Finally, we found that BCL2A1 and MCL1 were highly expressed in the dead group (Fig. 3E).

### A nomogram was created

**Fig. 4:**
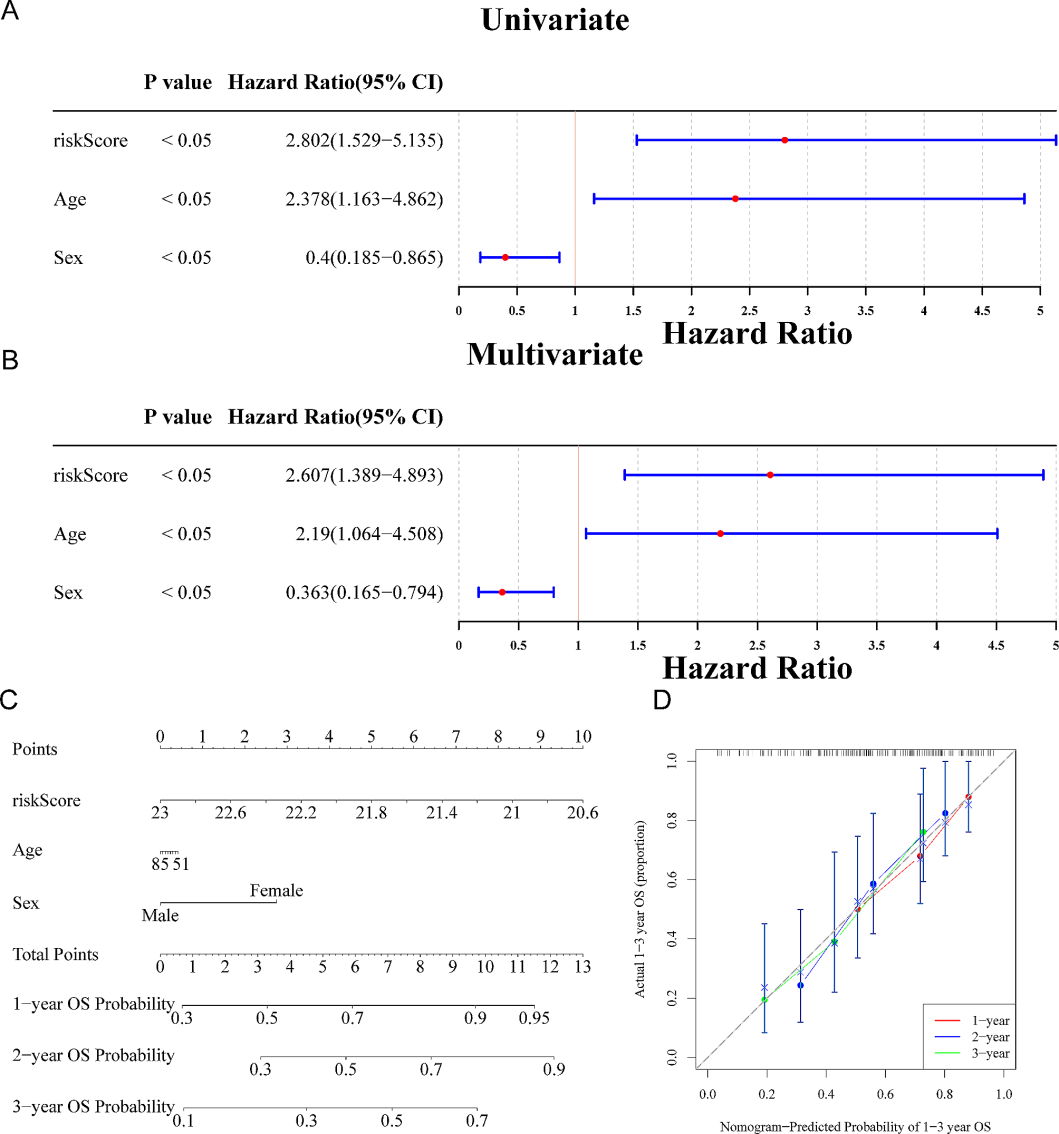
Construction of the nomogram model. **(A**-**B)** The forest plot of the univariate **(A)** and multivariate **(B)** Cox analyses of riskScore, Gender, and Age. (p-value < 0.05) **(C)** The nomogram model of riskScore, Gender, and Age in the GSE28042 dataset. **(D)** The calibration curve model to verify the predictive value of risk score regarding 1-, 2-, and 3-year survival

Independent prognostic analysis was employed to establish a nomogram. First of all, the riskScore, Gender, and Age were screened out by the univariate Cox analysis (*p* < 0.05) (Fig. 4A). Furthermore, the Age, riskScore, and Gender were achieved as independent prognostic factors (Fig. 4B). Accordingly, a nomogram for survival forecasting of IPF patients (1-, 2- and 3-year) was created based on those above independent prognostic factors (Fig. 4C). The calibration curve indicated that the nomogram had a high prediction accuracy (Fig. 4D).

### The GSEA and IPA based on the risk model

**Fig. 5:**
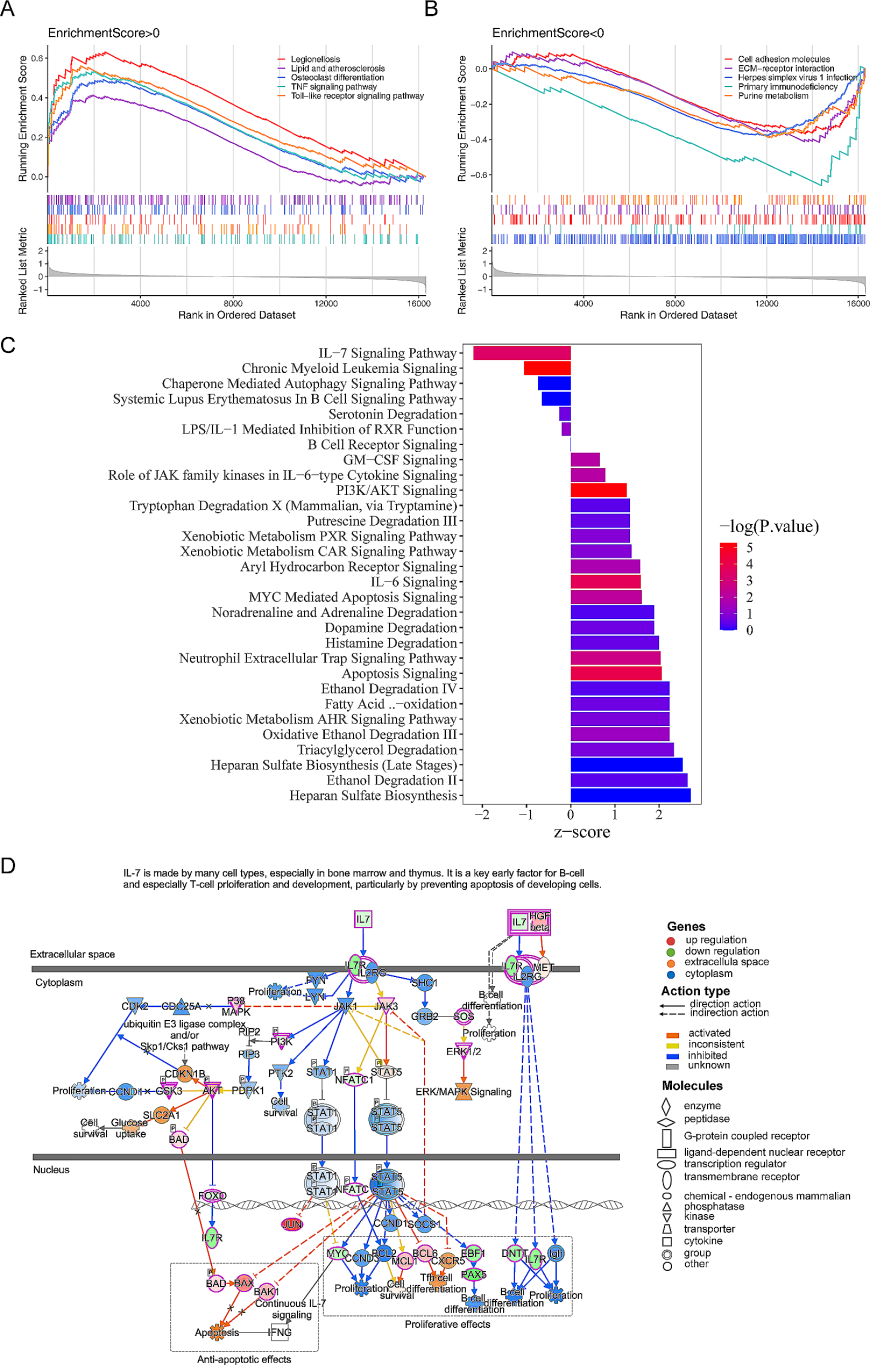
The Gene Set Enrichment Analysis (GSEA) and ingenuity pathway analysis (IPA) based on the risk model. (**A**-**B**) GSEA between high- and low-expression of the GSE28042 dataset. p-value < 0.05,|NES|>1) (**C**) The column plot of the 30 classical pathways. (**D**) The diagram of IL-7 signaling pathway transduction

We conducted the GSEA, and we found that some pathways such as ‘Osteoclast differentiation’, ‘TNF signaling pathway’, ‘Cell adhesion molecules’ and ‘Purine metabolism’ were enriched in those two risk subgroups (Fig. 5A-B, Supplementary Table 6). In addition, the IPA results showed that key genes were associated with 30 classical pathways including ‘Apoptosis Signaling’, ‘PI3K/AKT Signaling’, ‘IL-7 Signaling Pathway’, and so on, and it showed that the influence of key genes on signaling pathways was mostly promoting (Fig. 5C, Supplementary Table 7). The ‘IL-7 Signaling Pathway’ with the highest|z-score| (2.683) was selected to display, and we could find that ‘IL-7 Signaling Pathway’ regulated the growth, proliferation, and survival of immune cells by activating multiple signaling cascades such as JAK-STAT, PI3K-Akt and mTOR (Fig. 5D).

### The immune infiltration analysis between two risk subgroups

**Fig. 6:**
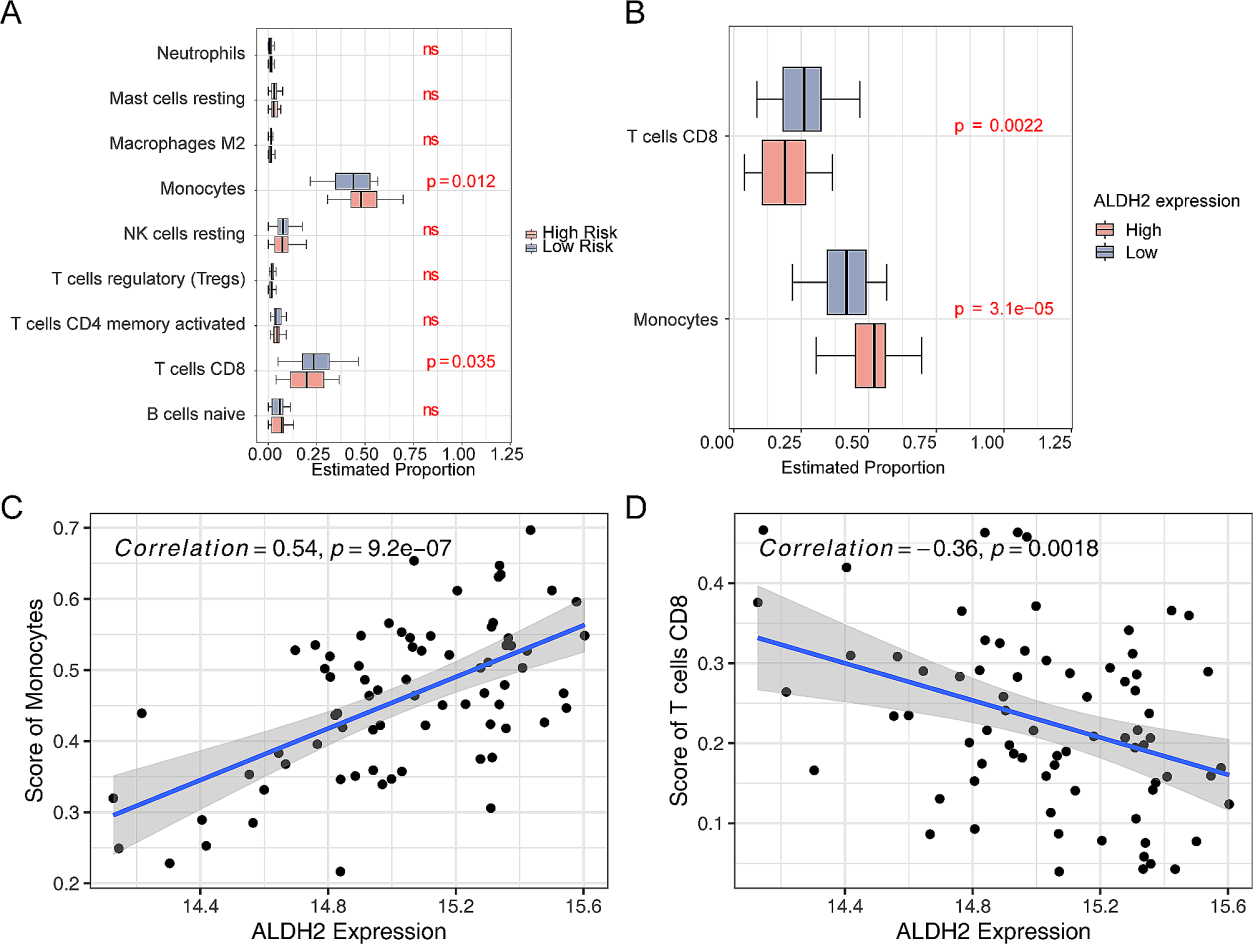
The immune infiltration analysis in high-risk and low-risk subgroups. (**A**) The abundance of 9 immune cells estimated by CIBERSORT algorithm in high-risk and low-risk groups in the GSE28042 dataset. (ns: no significance.) (**B**) The abundance of monocytes and CD8 T cells estimated by CIBERSORT algorithm in high-risk and low-risk groups based on the expression of *ALDH2* in the GSE28042 dataset. (p-value < 0.05) (**C**-**D**) The scatter plot of correlation analysis between *ALDH2* expression and abundance of monocytes and CD8 T cells. The gray area outside the slash indicates the 95% confidence interval of the slash

There were 2 differential immune cells including Monocytes and CD8 T cells between these two risk subgroups (Fig. 6A). Furthermore, two differential immune cells were all significantly different in the two expression subgroups of *ALDH2* (Fig. 6B). The Monocytes had a positively association with *ALDH2*, and the correlation coefficient was 0.54. Meanwhile, there was a negatively relationship between *ALDH2* and CD8 T cells (Cor = -0.36) (Fig. 6C-D).

### The lncRNA-miRNA-mRNA network and the biomarker-drug network were created

**Fig. 7:**
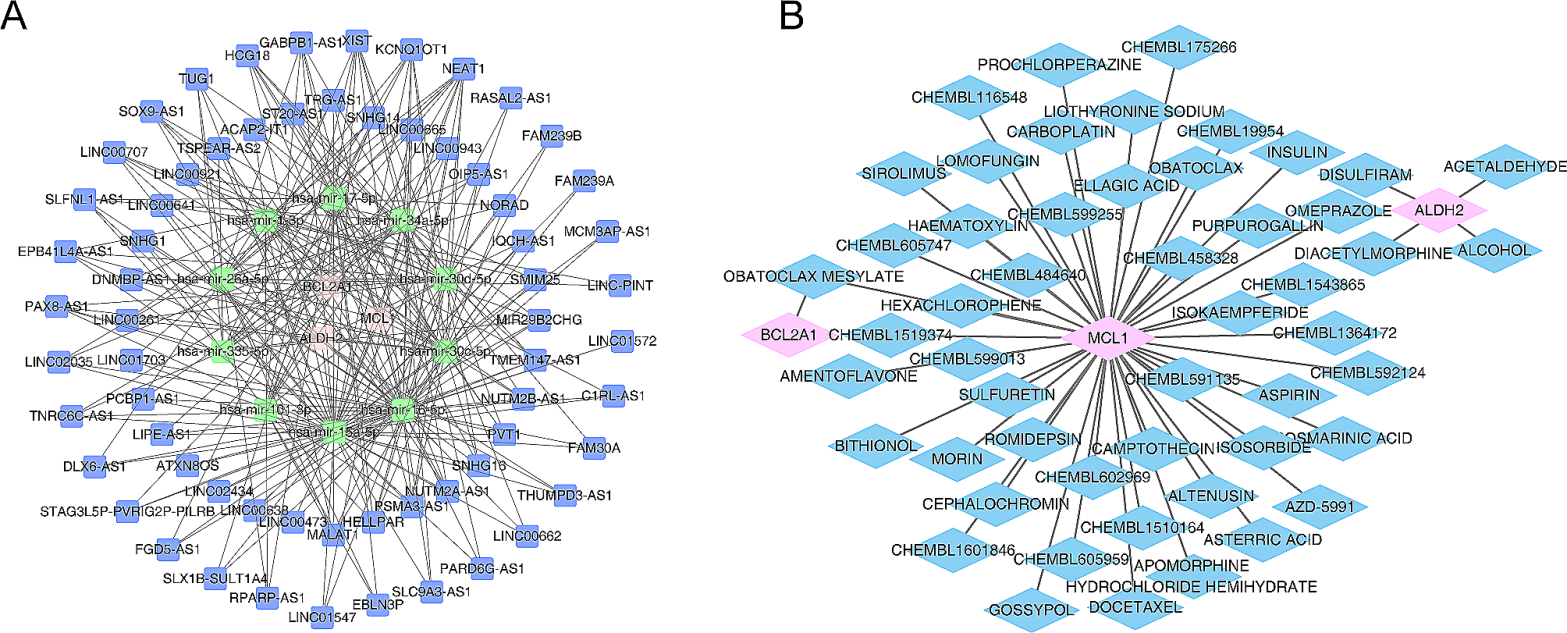
The bioinformatic analysis of molecular mechanisms. (**A**) The competing endogenous RNAs (ceRNA) of prognostic gene. Blue nodals represent long noncoding RNA (lncRNA), green nodals represent miRNAs, and red nodals represent mRNAs. (**B**) The network diagram of prognostic gene-drug. Blue nodals represent drugs and pink nodals represent prognostic genes

The lncRNA-miRNA-mRNA regulatory network was created in GSE28042, including 3 key genes, 10 miRNAs, and 64 lncRNAs. Further, we found that MIR29B2CHG and hsa-mir-1-3p could regulate the expression of *ALDH2*, and *MCL1*could be regulated by hsa-mir-15a-5p and XIST (Fig. 7A, Supplementary Table 8). Besides, the key genes -drug network was created, including “OBATOCLAX MESYLATE”, “SIROLIMUS”, “OMEPRAZOLE”, etc. (Fig. 7B, Supplementary Table 9).

### The expression levels of the key genes

**Fig. 8:**
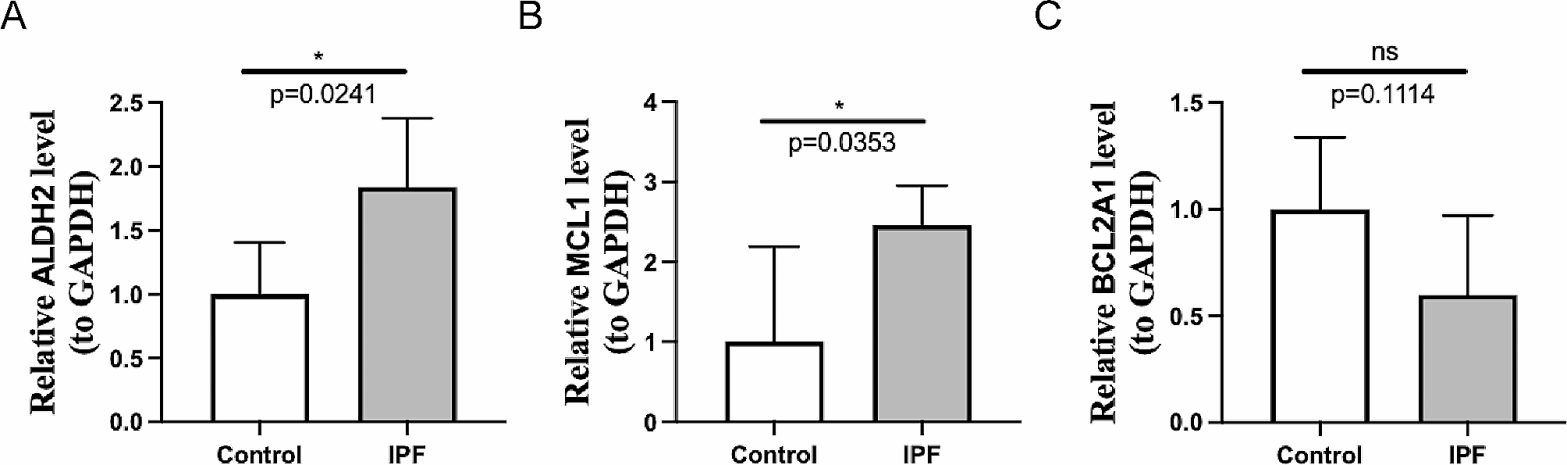
The Expression Levels of the key genes. (**A**-**C**) *ALDH2*, *MCL1* and *BCL2A1* expression in the IPF and control group (* *p* < 0.05, ns: no significance)

The RT-qPCR results indicated that the expression of the *ALDH2* and *MCL1* between IPF and control groups were markedly different. The *ALDH2* and *MCL1* were highly expressed in IPF (Fig. 8A-C). In summary, the results of RT-qPCR suggested that *ALDH2* and *MCL1* had good diagnostic value for IPF.

## Discussion

At present, the only anti-fibrosis drugs approved by the FDA are pirfenidone and Nidanib, both of which have obvious drug toxicity, and the treatment of IPF remains to be explored [7]. The median survival time after diagnosis of IPF is short, the prognosis is poor, and the mortality is high [2]. Mitochondria of IPF patients have different types of dysfunction. Macrophages regulate pulmonary fibrosis through various pathways such as NF-kB and PI3K-Akt-mTOR. Moreover, studies have shown that mitochondrial autophagy and apoptosis resistance of M2 macrophages are causally and causally involved in the process of IPF [14]. Mitochondria-related key genes may be able to predict the prognosis of IPF, and it is important for the treatment and prognosis prediction of IPF to construct a prognostic model by combining the study with macrophages.

Using univariate cox regression to screen 26 shared genes, three genes, *ALDH2*, *MCL1*, and *BCL2A1*,were found to be significantly associated with survival. The risk ratio of all genes significantly associated with survival was greater than 1, indicating that they were all risk factors promoting disease development. *ALDH2* gene (aldehyde dehydrogenase 2), located on human chromosome 12, is a key mitochondrial enzyme in the metabolism of acetaldehyde and is closely related to oxidative stress [30, 31]. *ALDH2* gene enrichment in Alcoholic liver disease, Pantothenate and CoA biosynthesis pathways. The Alcoholic liver disease pathway is closely related to oxidative stress [32], and the Pantothenate and CoA biosynthesis pathways are closely related to lipid, protein and other compounds and energy metabolism [33]. High expression of *ALDH2* can reduce the expression of fibrotic genes and excessive deposition of extracellular matrix in fibroblasts [34]. *ALDH2* deficiency can cause mitochondrial biogenesis disorder of cardiomyocytes [35]. The upregulation of *ALDH2* can inhibit myocardial cell damage induced by high glucose and alleviate myocardial fibrosis in rats. The mechanism of action may be related to oxidative stress and inflammation involved in MMP14 and TIMP4 [36]. In addition, upregulation of *ALDH2* can regulate autophagy to protect renal tubular epithelial cells through the Beclin-1 pathway [37], thereby ameliorating acute kidney injury and neuronal damage caused by hypoxia [38]. Both the *MCL-1* gene and *BCL2A1* gene are derived from the *BCL-2* gene family. *MCL-1* gene is one of the anti-apoptotic proteins, which can be targeted to induce mitochondrial autophagy and enriched in the Apoptosis Signaling pathway [39]. *MCL-1* is overexpressed in breast cancer, lung cancer and other cancers, and is associated with resistance to chemotherapy drugs [40]. *BCL2A1* is enriched in Apoptosis Signaling and NF-kappa B signaling pathway. Studies have shown that *BCL2A1* can inhibit TNF-α-induced endothelial cell damage and reduce cell death through the NF-κB pathway [41]. In addition, *BCL2A1*is involved in advanced metastasis of ovarian cancer, breast cancer, acute and chronic leukemia and other blood diseases and cancers [42]. In addition, TNF released by M1 macrophages changes the phenotype of macrophages and fibroblasts, delays tissue repair, and also produces TGF-β1 and platelet-derived growth factor (PDGF), promoting pulmonary fibrosis [43]. Therefore, it is speculated that the profibrotic effect of *ALDH2* gene is related to oxidative stress and energy metabolism. In addition, this study found for the first time that *MCL-1* and *BCL2A1* genes may play an important role in the occurrence and development of IPF, and both genes are enriched in the Apoptosis Signaling pathway. We speculate that the mechanism of MCL-1 and *BCL2A1* participating in promoting pulmonary fibrosis is related to apoptosis resistance. But the specific functional mechanism needs to be further studied.

We found that monocytes and CD8 T cells were different between the high and low risk groups of IPF. There were significant differences in *ALDH2* gene expression between the two types of immune cells, but no significant differences in *MCL-1* and *BCL2A1* expression between the two groups. Monocytes are derived from hematopoietic stem cells in bone marrow and can differentiate into macrophages and myeloid dendritic cells. A retrospective analysis in 2021 indicated that monocyte count was positively correlated with the number of acute exacerbations and mortality in patients with IPF, and the blood routine was simple and convenient, so monocytes could be a promising serum biomarker for the prognosis of IPF [44]. CD8 T is a key component of the adaptive immune system that monitors the body and clears infections. Bleomycin was injected into both IL-21 receptor deficient mice and wild mice, but collagen deposition and α-smooth muscle actin expression were significantly reduced in lung tissue of IL-21 receptor deficient mice. IL-21 is a key cytokine for differentiation of CD8 T into Tc2 cells, demonstrating that CD8 T is involved in pulmonary fibrosis in an IL-21-dependent manner [45]. We analyzed the differences in immune cells between *ALDH2* expression subgroups and found that *ALDH2* was positively correlated with monocytes and negatively correlated with CD8 T cells. Studies have shown that people with *ALDH2* genotype (*ALDH2* *1/*2 and ADH1B*2) are more likely to have damage to the number and function of peripheral blood monocytes after receiving alcohol [46], which is different from our analysis results, and may be related to the existence of monocytes in peripheral blood or differentiation into macrophages. In summary, the occurrence and development of IPF may be closely related to monocytes and CD8 T cells.

We further made potential drug prediction of key genes, and found that Obatoclax is a hydrophobic small molecule inhibitor of the BCL-2 family, which mainly acts on tumors, respiratory diseases, and immune system diseases [47]. The *MCL-1* gene and *BCL2A1* gene that can promote fibrosis are derived from the BCL-2 family. Obatoclax may play an anti-fibrosis role by regulating cell apoptosis resistance. Docetaxel is a plant-derived drug that exerts anti-tumor effects by inducing cell cycle arrest and is closely related to CD8 T cells [48]. Docetaxel may be associated with IPF via CD8 T cells and the *ALDH2* gene. There are also Sirolimus, Aspirin, Prochlorazine, Disulfiram, Romidepsin and Omeprazole 6 drugs. Sirolimus is a specific mTOR inhibitor used to suppress the function of the human immune system [49]. Aspirin is mainly used as antipyretic and analgesic drugs, non-steroidal anti-inflammatory drugs, anti-platelet aggregation drugs [50]. Prochlorperazine is a phenothiazine drug with a piperazine side chain, which has antipsychotic effects [51]. Disulfiram acts primarily by irreversibly inhibiting intracytoplasmic and intracmitochondrial acetaldehyde dehydrogenase in the treatment of alcohol-dependent and cooperative alcoholics [52]. Romidepsin is a histone deacetylase (HDAC) inhibitor with antitumor activity for cutaneous T-cell lymphoma [53]. Omeprazole is a first-generation proton pump inhibitor, which can inhibit gastric acid secretion and protect the stomach mucosa [54]. The above-mentioned drugs have been widely used in other fields, but the anti-fibrotic effect still needs further study.

A risk model for predicting the prognosis of IPF was developed using univariate Cox analysis and Lasso regression analyses. The model was validated using data from the GSE27957 dataset, and ROC curves showed that the three key genes obtained were able to predict poor prognosis in IPF patients. A nomogram was constructed to visualize the 1 to 3 year overall survival (OS) rates, demonstrating that our model has clinical advantages and performs well in predicting outcomes. Yu Li et al. constructed a prognostic model in the GSE28042 dataset. By comparison, we found that our results showed better AUC values at 1-year and 2-year time points, indicating a more accurate prediction of patient prognosis. Additionally, Lu Y et al. also constructed a prognostic model in GSE28042 similar to ours, further validating the superior performance of our model [55, 56]. 

Validation of the prediction model using mitochondria and macrophagerelated genes for IPF in a clinical patient population is crucial to ensure its generalizability and applicability in real-world settings. The sample size in this study was relatively small, underscoring the need for future research with larger sample sizes to improve the reliability and robustness of the predictive model. As this study relied on retrospective data analysis, conducting prospective studies is necessary to validate and corroborate the findings in an independent cohort. Furthermore, given the substantial size of the MaRGs gene set, the identified genes are inclined towards mitochondrial functions. Consequently, additional delineation is essential to elucidate the interplay between MaRGs, MitoRGs, and IPF DEGs in future investigations.

## Conclusions

IPF is an irreversible chronic lung disease with poor prognosis, poor quality of life and short survival time. Its course and survival rate are difficult to predict. In this study, clinical and biological characteristics of patients were analyzed and comprehensively evaluated. The intersection of IPF differentially expressed genes, macrophage related genes and mitochondria related genes was combined with database analysis to obtain *ALDH2*, *MCL1*, and *BCL2A1*genes, and it was speculated that the mechanism of their participation in IPF was related to oxidative stress, energy metabolism and cell apoptosis resistance. Moreover, a prognostic model of IPF was constructed to identify two related immune cells, monocyte and CD8T, and to predict potential effective drugs. However, the specific experimental mechanism needs to be further studied, and we will continue to pay attention to the research progress of 13 genes *ALDH2*, *MCL1*, and *BCL2A1*in the future. In conclusion, this study fills the gap in prognostic key genes of IPF, can better predict the disease progression and prognosis of patients with IPF, and promote the development and progress of clinical research.

### Electronic supplementary material

Below is the link to the electronic supplementary material.

Supplementary Material 1: The clinical characteristics of the IPF patients in GSE27957.

Supplementary Material 2: The clinical characteristics of the IPF patients in GSE28042.

Supplementary Material 3: Differentially expressed genes (DEGs) between Idiopathic Pulmonary Fibrosis (IPF) and Control groups.

Supplementary Material 4: The 307 Gene Ontology (GO) Biological Process (BP) terms, 14 GO Cellular Components (CC) terms, and 12 GO Molecular Function (MF) terms were enriched for 26 signature genes.

Supplementary Material 5: The 50 Kyoto Encyclopedia of Genes and Genomes (KEGG) pathways were enriched for 26 signature genes.

Supplementary Material 6: The 41 Gene Set Enrichment Analysis (GSEA) pathways in the GSE28042 dataset.

Supplementary Material 7: The 30 classical pathways of the column plot of the 30 classical pathways in the GSE28042 dataset.

Supplementary Material 8: The 268 pairs of regulatory relationships in the competing endogenous RNAs (ceRNA) regulatory network.

Supplementary Material 9: The 52 pairs relationships in the predictive network of prognostic genes and drugs.

## Data Availability

The GSE28042 dataset and the GSE27957 dataset analysed during the current study are available in the Gene Expression Omnibus (GEO) database (https://www.ncbi.nlm.nih.gov/gds). 1136 Mitochondria-related genes (MitoRGs) analysed during the current study are available in the MitoCarta3.0 online database (http://www.broadinstitute.org/mitocarta). 3201 Macrophage-related genes (MaRGs) analysed during the current study are available in the GeneCards online database (https://www.genecards.org/).

## Abbreviations

IPF: Idiopathic Pulmonary Fibrosis
EMT: epithelial-mesenchymal transformation
TAFs: telomere-associated DDR sites
GEO: Gene Expression Omnibus
PBMC: peripheral blood mononuclear cell
MitoRGs: Mitochondria-related genes
MaRGs: Macrophage-related genes
GO: Gene Ontology
KEGG: Kyoto Encyclopedia of Genes and Genomes
PPI: Protein Protein Interaction
LASSO: least absolute shrinkage and selection operator
K-M: Kaplan-Meier
GSEA: Gene Set Enrichment Analysis
IPA: ingenuity pathway analysis
lncRNA: long noncoding RNA
AUC: area under curve
PDGF: platelet-derived growth factor
HDAC: histone deacetylase
